# KB220Z™ a Pro-Dopamine Regulator Associated with the Protracted, Alleviation of Terrifying Lucid Dreams. Can We Infer Neuroplasticity-induced Changes in the Reward Circuit?

**DOI:** 10.17756/jrdsas.2016-022

**Published:** 2016-05-19

**Authors:** Thomas McLaughlin, Marcelo Febo, Rajendra D. Badgaiyan, Debmalya Barh, Kristina Dushaj, Eric R. Braverman, Mona Li, Margaret A. Madigan, Kenneth Blum

**Affiliations:** 1 Center for Psychiatric Medicine, North Andover, MA, USA; 2 Department of Psychiatry and McKnight Brain Institute, University of Florida, College of Medicine, Gainesville, FL, USA; 3 Molecular and Functional Imaging Laboratory, Department of Psychiatry, University of Minnesota, Minneapolis, MN, USA; Neuromodulation Program, University of Minnesota Twin City Campus, Minneapolis, MN, USA; Laboratory of Advanced Radiochemistry, University of Minnesota Twin City Campus, Minneapolis, MN, USA; 4 Centre for Genomics and Applied Gene Technology, Institute of Integrative Omics and Applied Biotechnology (IIOAB), Nonakuri, Purba Medinipur, India; 5 Department of Clinical Neurology, PATH Foundation NY, New York, NY, USA; 6 Department of Personalized Medicine, IGENE, LLC., Austin, TX, USA; 7 Community Mental Health Institute, Center for Clinical & Translational Science, University of Vermont and Department of Psychiatry, University of Vermont College of Medicine, Burlington, VT, USA; 8 Division of Addiction Services, Dominion Diagnostics, LLC., North Kingstown, RI, USA; 9 Division of Neuroscience-Based Therapy, Summit Estate Recovery Center, Los Gatos, CA, USA; 10 Department of Nutrigenomics, LaVita RDS, LLC, Salt Lake City, UT, USA; 11 Division of Neuroscience Research & Addiction Therapy, Shores Treatment & Recovery Center, Port Saint Lucie, FL, USA; 12 Department of Education & Psychology, Eotvus Lorand University, Budapest, Hungary

**Keywords:** Dopamine, Lucid dreams, Neuroplasticity, Connectivity volume, KB220z

## Abstract

**Background:**

Recent reports by our laboratory have indicated that lucid dreams may be linked to psychiatric conditions, including Attention Deficit Hyperactivity Disorder (ADHD) and other Reward Deficiency Syndrome-related diagnoses. In the latter case, it has been our observation that such lucid dreams can be unpleasant and frequently terrifying.

**Case presentations:**

We present four cases of a dramatic and persistent alleviation of terrifying, lucid dreams in patients diagnosed with ADHD/PTSD and/or opiate/opioid addiction. The amelioration of such dreams could well be permanent, since the patients had stopped taking the nutraceutical for between 10 to 12 months, without their recollection or recurrence. In the first case, the patient is a 47-year-old, married male who required continued Buprenorphine/ Naloxone (Suboxone) treatment. The second case involved a 32-year-old female with the sole diagnosis of ADHD. The third case involves a 38-year-old male who carried the diagnoses of Substance Use Dependence and ADHD. The fourth case involved a 50-year-old female with the diagnoses of Alcohol Abuse, ADHD and Posttraumatic Stress Disorder.

**Results:**

In order to attempt to understand the possibility of neuroplasticity, we evaluated the effect of KB220Z in non-opioid-addicted rats utilizing functional Magnetic Resonance Imaging methodology. While we cannot make a definitive claim because rat brain functional connectivity may not be exactly the same as humans, it does provide some interesting clues. We did find following seeding of the dorsal hippocampus, enhanced connectivity volume across several Regions of Interest (ROI), with the exception of the pre- frontal cortex. Interestingly, the latter region is only infrequently activated in lucid human dreaming, when the dreamer reports that he/she had the thought that they were dreaming during the lucid dream.

**Conclusions:**

The four patients initially reported a gradual but, then, complete amelioration of their long-term, terrifying, lucid dreams, while taking KB220Z. The persistent amelioration of these dreams continued for up to 12 months, after a self-initiated, cessation of use of KB220Z. These particular cases raise the scientific possibility that KB200Z increases both dopamine stability as well as functional connectivity between networks of brain reward circuitry in both rodents and humans. The increase in connectivity volume in rodents suggest the induction of neuroplasticity changes, which may be analogous to those involved in human lucid dreaming as well as Rapid Eye Movement sleep. The possibility that the complex induces long-term, neuroplasticity changes must await more intensive investigations, involving large-population, double-blinded studies.

## Introduction

Lucid dreams are a type of imaginary life, during which the dreamer may be aware that he/she is dreaming, can voluntarily end or re-start the dreams, depending on their content, and be aware that his lucid dream is, in fact, a dream. Lucid dreams appear to be associated with a variety of psychiatric conditions, including Posttraumatic Stress Disorder (PTSD), Attention Deficit Hyperactivity Disorder (ADHD), and other Reward Deficiency Syndrome (RDS) associated diagnoses. In these clinical conditions, lucid dreams may assume an unpleasant and frequently, terrifying character. In our previous papers, we characterized the role of neurotransmitters in lucid dreams [1, 2].

There is no scientific consensus regarding the mechanism(s) of nightmares, their function, and what their neuropsychological triggers might be. One model proposes a role for the hippocampus, and relates this to the disruption of contextual memory by dreams, occurring during paradoxical Rapid Eye Movement (REM) sleep. The neurochemical mechanisms have been postulated by many investigators and can be reviewed [3], by which neurotransmitters such as acetylcholine, cortisol and dopamine may be elevated, whereby serotonin and norepinephrine activation may be absent.

A PubMed search (3-1-16) listed 587 nightmares and 6 lucid dreams. Bowirrat et al. [4] pointed out that trauma, especially during early life, have lasting results on neurochemical reactions to later stressful occurrences. These outcomes involve the degree of catecholamines (dopamine/ norepinephrine) response as well as the duration and extent of the cortisol response as well as phasic dopamine tone, especially in ADHD patients [5]. Understanding lucid dreams in RDS and associated subsets such as PTSD and trauma is quite complex, and many investigators have attempted to unravel these complexities [6–14]. Interestingly, others have proposed that synaptic plasticity at glutamate synapses throughout the brain play a part in neurodevelopmental as well as a range of mature neural functions [15]. It has been recently demonstrated that the developmental disorder, ADHD, is associated with dysregulated dopamine neurotransmission [5]. This study found that the tonic pool of dopamine is attenuated, and the phasic release is enhanced in this condition. Dysregulated dopamine, therefore, appears to be equally disruptive to normal mechanisms of synaptic plasticity. In PTSD, however, long-term potentiation is necessary for conditioned fear extinction and is thought to be impaired because of reduced dopamine release during the processing of emotional memory as proposed by Kwon et al. [16]. Moreover, in PTSD, there is evidence of a diminution in overall brain size as well as in the gray and white matter of numerous areas, most notably, the dorsolateral and ventromedial prefrontal cortex, but also the hippocampus, amygdala, and corpus callosum (CC). Diffusion tensor imaging (DTI) reports indicate evidence of disruption in inter-regional connectivity between these regions [17]. There are many studies that suggest a direct association between childhood abuse and brain measures of volume and connectivity, indicating that the most obvious disruptions linked to early childhood abuse occur in the function and structure of lateral and ventromedial fronto-limbic brain regions and systems that facilitate behavior and influence such behaviors of sleep and working memory [18, 19].

Previously, the authors reported the clinical effects of KB200Z in reducing the frequency of terrifying lucid dreams. Specifically, we described two cases, who following the administration of KB220Z, had their terrifying lucid dreams converted to ones full of happiness, nostalgia and laughter [1]. In a second study, McLaughlin et al. [2] reported eight additional cases with histories of opioid substance abuse, and childhood abuse, which was also diagnosed with PTSD and RDS-related diagnoses. In this study, the administration of KB200Z was associated with the cessation of unpleasant, frightening, lucid dreams in 87.5% of the cases, with one former heavy cocaine user (greater than 10,000 hits) reporting only a minimal response, possibly due to reduced dopamine neuronal stores [2].

Remarkably, in this current study, four individuals carrying a diagnosis with or without opioid dependence, with a comorbid diagnosis of ADHD and PTSD showed a pronounced effect on the frequency of terrifying lucid dreams that occurred and complete elimination of the lucid dreams resulting after they stopped using KB200Z. This finding prompted this report because it may have important relevance to this well-deserved field involving terrifying dreams following traumatic experiences in childhood.

We the authors believe that the above mechanistic speculations could ultimately suggest that dopamine agonist therapy (potentially using a pro-dopamine regulator like KB220Z) may be both a preventative as well as a therapeutic intervention for lucid dreams associated RDS diagnoses, such as ADHD. Moreover, while not definitively linked to humans, our rat fMRI study may provide some clues to potentially understanding the protracted effect of KB220Z [20, 21]. The rationale for the rat study is not to suggest that rats are having lucid dreams like humans but to determine if there is an increase in connectivity volume following a single dose of KB220z. If an increase in connectivity is indeed found, this could suggest a mechanism for neuroplasticity. We have reported earlier in abstinent heroin addicts that KB220Z using fMRI increased brain functional connectivity [7].

The present case study provides some evidence that KB220Z complex may have resulted in a long-term, and possibly, permanent elimination of the frequent recurrent, terrifying lucid dreams. It is hereby hypothesized that this noted effect may have been achieved through the induction of enhanced connectivity volume in selected brain regions thought to be involved in the genesis of lucid dreams.

The ingredients of KB220Z involve various precursor amino-acids for synthesis of serotonin, including a chromium salt; L-Glutamine for gamma-aminobutyric acid (GABA) and L-Phenylalanine and L-Tyrosine for dopamine; D-Phenylalanine, a recognized essential enkephalinase inhibitor; Passion Flower, a natural benzodiazepine excitant and an herbal ingredient (*Rhodiola rosea*) identified as an inhibitor of Catechol-O-methyltransferase (COMT) and mitochondrial Monoamine oxidase A (MAO-A) [22].

## Presentation of Cases

### Case one (Suboxone patient) OB

#### History

The patient is a 47-year-old, married male, who presented with a chief complaint of needing to transfer continuation of his buprenorphine/naloxone (Suboxone) treatment. He had been treated with two Suboxone 8 mg / 2 mg tablets a day for seven years, with occasional relapses. He stated he had also been diagnosed with ADHD and was seeking treatment for this condition as well.

#### Past psychiatric history

The patient denied any history of long-term psychotherapy or psychiatric hospitalizations.

#### Family psychiatric history

The patient denied any family history of major psychiatric illness.

#### Family history

The patient stated his mother was 65-years-old and was neither attentive nor affectionate to him as a child. He reports his father was in his late 60’s and also had not been attentive or affectionate. The patient noted that his mother would walk in front of the children and family semi-naked (Exhibitionism is noted by Blum et al. [6] to be a manifestation of Reward Deficiency Syndrome).

#### Past medical history

The patient denies any history of any major medical problems including hepatitis, coronary artery disease, hypertension, diabetes, and thyroid disease.

#### Abuse history

The patient denied any history of sexual, verbal or physical abuse.

#### ADHD history

The patient reports he was easily distracted in grade school and was also quite restless. He admitted to vocal and motor tics. His concentration had been extremely poor and he was and is unable to focus his attention easily or read books. He rated his tendency towards impulsivity and procrastination as “high” and also endorsed behaviors and thoughts consistent with obsessive-compulsive disorder (OCD).

#### Substance use history

The patient first used marijuana at age 12; snorted cocaine at age 15; and began using Percocet, Vicodin and OxyContin at age 27. He had been using OxyContin at a cost of $400 a day. When he used substances, he felt “on top of my game.” He also reported a history of heroin use and had used cocaine approximately 5,000 times over a period of 15 years. He also used Ecstasy for ten years, starting at age 12 and reported a smoking addiction, involving more than two packs a day for twenty years.

#### Mental status examination

The patient was alert and oriented in all three spheres. He was pleasant and cooperative, with no evidence of suicidal or homicidal ideation. There was also no evidence of mood changes, including no history of mania. He denied current or past hallucinations or delusions. Appetite was normal, but sleep was disturbed as noted below.

#### Dream life

The patient reported a history of vivid, lucid, terrifying dreams (“80% are unpleasant”). After one month’s use of KB220Z, 2 tablets in the morning and 2 tablets in the evening, the frequency of his unpleasant dreams had decreased from nightly to two-three times a week. During the next month’s visit, the patient reported some increased cravings, but no use of opiates. His Suboxone was increased to 2½ pills a day, and he was referred to AA. There was a 12-month hiatus in his treatment. When next seen, he reported some cravings, but no opiate use. He was still smoking two packs of cigarettes a day.

During his next visit, he reported no cravings and no use. Two weeks, later he continued not to have cravings and no use of opiates. During his next visit, the patient reported decreased cravings and no use of opiates. He had lost 38 pounds over the prior six months. He had been treated for ADHD by another clinician. At this point, he stated his dreams, which were still lucid and life-like in nature, were about 80% “pleasant.”

During his next visit, he reported decreased cravings and no use of opioids. His lucid dreams on KB220Z had now decreased to two to three times a week and were noted as “80% pleasant.” During his next visit, he reported decreased cravings and no use of opiates. He was euthymic, and his concentration was good. His dreams were now uniformly “pleasant” and no longer “lucid and life-like.” During his next visit, he stated he had no cravings and no use. He complained of some low energy of a physical nature. He was sleeping and eating normally, however. He reported some stress, secondary to marital problems. He stated that his lucid dreams were now occurring “only occasionally,” but were somewhat terrifying in nature. During his next visit, he reported no cravings and no use. His mood was euthymic. He was taking only one KB220Z a day and could no longer remember any dreams. Seven months later he was evaluated again, having had different providers in the meantime. He denied cravings for opiates and reported no relapse. His mood was stable, without any stressors. During this visit, he reported he had only been taking two KB220Z a day for four months and had eventually stopped the KB220Z completely for the three months prior to this visit. He specified that he had not had any vivid, lucid dreams of either a terrifying or pleasant nature, since stopping the KB220Z. He later reported he had not had any lucid, terrifying dreams for over 10 months.

### Case two (Suboxone patient) JC

#### History

The patient is a 32-year-old, single woman, with no children. She was referred for the treatment of opiate addiction. She stated: “I’ve been addicted to Vicodin and Percocet, since age 20 or 21, and I’m now 32; I had lost everything - my job, my friendships, my relationships and even, myself.” The treatment plan was to continue the patient on her Suboxone 8 mg / 2 mg twice a day, a regimen she had been treated with successfully for three years.

#### Past psychiatric history

With the exception of addiction treatment, she denied any history of psychiatric treatment or hospitalizations.

#### Family psychiatric history

The patient denied any formal psychiatric diagnoses in her immediate family.

#### Family history

The patient’s father is a 54-year-old construction worker. She has had no contact with him for ten years. Her mother is 52-years-old. She described her mother as “mean.”

#### Social history

The patient had an 11^th^ grade education, after which she obtained a GED. She later attended a local community college for one year. She then worked as an employee at a local department store, which she managed for eleven years. Currently, she works at a local psychiatric hospital as a counselor. She has never married and has no children.

#### Abuse history

The patient denied any history of sexual or physical abuse, but reported that her mother had been verbally and emotionally abusive. She implied significant, emotional conflicts with her father (from whom she was estranged), but refused to elaborate.

#### ADHD history

During grade school, the patient stated that she was dreamy, spacey, and unable to focus. She was not a “Chatty Kathy,” nor was she restless. She was frequently bored.

#### Substance abuse history

The patient reported a 12-year history of addiction to opiates (see above).

#### Mental status examination

The patient was alert and oriented in all three spheres. She was pleasant and cooperative and denied depressed mood, suicidal or homicidal ideation, psychotic symptoms or any history of mania, OCD, panic symptoms, or appetite disturbance. Her sleep pattern is described below.

#### Dream life

The patient reported the occurrence of vivid, lucid dreams of a terrifying nature. Her dreams would frequently involve fires, enveloping her, or dreams of being murdered, chased, or stabbed. Her lucid, terrifying dreams began at five years of age and have occurred about five times a week, until the present. Their content has been almost uniformly “unpleasant and scary.” She reported she was able to control these dreams at times and was sometimes aware that she was dreaming. Like some of the patients in one of the authors’ (TM) experience, she was frequently afraid to fall asleep because of the terrifying nature of her dreams. She added that the dream of being chased during her nighttime dreams often led to a non-psychotic “paranoia during the daytime.”

#### Treatment course

For scheduling reasons, subsequent Suboxone visits were divided between two providers. Because of one the authors prior experience and published reports [1, 2], in addition to her Suboxone, the patient was started on KB220Z, two tablets in the AM and two tablets in the PM, on an empty stomach.

For all of her subsequent visits, she continued on Suboxone and remained free of relapse and cravings. Occasionally, she reported job-related stress. She subsequently admitted to only taking two tablets a day for the first two months after KB220Z was initiated. After two months of taking half the recommended dosage, the patient reported that her vivid, lucid, terrifying dreams had completely stopped. During her most recent visit, which occurred ten months after her KB220Z was initially started, the patient reported that she had stopped her KB220Z two months after beginning it because her vivid, lucid, terrifying dreams had ceased. She stated that she had not experienced any vivid, lucid, or terrifying dreams for a total of 12 months, six of which while being completely off KB220Z.

### Case three (Suboxone patient) HK

#### History

The patient is a 38-year-old male that was originally referred for treatment of his opioid dependence. He reported a longstanding problem with Percocet and later, OxyContin. He had used the latter daily, until seven or eight months prior to admission to the Suboxone clinic. He reported occasional marijuana use. Since Suboxone treatment was initiated five years prior, he has had no side effects or problems with relapses or cravings. He was being maintained on one Suboxone 8 mg / 2 mg, sublingual tablets, three times a day.

#### Past psychiatric history

The patient had attended family counseling for about eight months in the distant past. He has not had any psychiatric hospitalizations.

#### Family psychiatric history

The patient denied any family history of psychiatric treatment.

#### Family history

The patient is an only child. He states his parents live together, and he had no problems during his childhood, with respect to abuse or neglect. There was no history of psychiatric illness or substance abuse in his family.

#### Abuse history

The patient denied any history of sexual, physical, or verbal/emotional abuse as a child.

#### Social history

The patient is married and has no children. He has a high school education and is employed as a landscaper.

#### Past medical history

The patient denied any history of significant medical problems including hepatitis, cardiovascular disease, hypertension, asthma, or seizures. He also denied any allergies to food or medications.

#### ADHD history

He reported concentration problems and problems with boredom and procrastination since childhood. He agreed to be started on Adderall XR 10 mg with upward titration.

#### Substance abuse history

The patient reported having used cocaine on occasion, but stated his drug of choice was OxyContin, which he had used for five years prior to entering the Suboxone program. He had never used intravenous cocaine or heroin.

#### Mental status examination

When initially seen, he was casually dressed and made good eye contact. He was oriented in all three spheres and was cooperative and pleasant. He appeared motivated for treatment. His thought processes were organized and linear. He denied any suicidal or homicidal ideation. He also denied auditory hallucinations or other psychotic symptoms. His appetite was normal and his sleep was disturbed, as noted below.

#### Dream life

With respect to his dream life, he reported vivid, lucid dreams, which were unpleasant “50% of the time” and occurred about twice a week. He stated they began in childhood and had not changed in any way, with the addition of Adderall to his regimen. He also reported a history of sleep paralysis.

#### Treatment course

When initially seen by one of the authors (TM) four years into treatment, he denied cravings or drug use. At his next visit, he denied any use of opiates or cravings. Because of his complaint of a history of vivid, lucid, terrifying dreams, the patient was started on KB220Z, two tablets in the morning and two in the evening, on an empty stomach.

One month later, the patient stated he had had no cravings or use of opiates. He was now having vivid, lucid dreams on rare occasions. During his next visit, he continued to report success with the Suboxone treatment, even though he had been recently laid off and was collecting unemployment.

One month later, he reported that he was still sober, euthymic and still collecting unemployment. During his next visit, his mood was normal and he had no cravings or drug use. He was now working regularly. One month later, the patient reported no cravings or use of opiates. He was planning to get married the next day.

Two months later, he reported no cravings or use of opiates. His concentration had improved on Adderall 10 mg, but he did not want to increase it. During his next visit, he was taking KB220Z, four tablets twice a day (i.e., twice the typical dose). He reported an increased sense of energy with the addition of KB220Z compared to Suboxone alone. He stated that his dreams had become “unusually happy” and he felt that his overall energy level and mood were “back to normal” (i.e., the way they were before he started Suboxone). During the next visit, his mood was euthymic and he had no cravings or any use. He stated that his concentration continued to be improved. During his next visit, the patient reported continued abstinence. He reported that he was now having “happy dreams as well as increased energy.” This result was on KB220Z, four tablets in the morning and four at bedtime. Sleep was otherwise normal. His concentration remained “good.”

During the next three visits, he reported no cravings or use and no major stressors. During the subsequent visit, he reported no cravings or relapse and no major stressors at home. At this point, he reported that he had not had any vivid, lucid dreams for more than three months and had stopped KB220Z three months prior to this visit. During the next visit, he maintained that his mood was good, and he had not used any opiates nor did he have any cravings. During the most recent visit, he stated he had not used opiates and did not have any cravings. At this point, he reported he had stopped the KB220Z for ten months. He had no dreams he could recall that were lucid or terrifying for the past five months.

### Case four AC

#### History

AC was a 55-year-old, divorced woman, living with her daughter. Her chief complaint was unremitting depression and persistent suicidal ideation. Axis 1 diagnosis revealed PTSD; Major Depressive Disorder (MDD); ADHD, inattentive type; Axis II: N/A; Axis III: obesity, s/p thyroidectomy; Axis IV: severe – symptom management; Axis V: CGAF 40.

#### Past psychiatric history

The patient had been in psychotherapy for four years, but never discussed any childhood or marital abuse. She has had four psychiatric hospitalizations. The patient attempted suicide three times, once involving crashing her motorcycle into a bus.

#### Abuse history

The patient was sexually abused by two uncles, involving sexual intercourse as well as by a cousin during her childhood. With respect to physical abuse, there was extreme violence in the home and her mother was frequently beaten by her father. She herself was beaten at five years of age by her father and never told her mother. She was emotionally abused by her cousins. The patient was the victim of physical violence by her first husband and verbal abuse by her second husband.

#### Family psychiatric history

There were numerous suicides in the family. Her father was an alcoholic. (Later, it was determined that the father had presumed Tourette’s Syndrome, in view of his frequent throat-clearing and motor mannerisms). Her sexually abusive uncles both also had prominent vocal tics.

#### Family history

The patient’s mother is 75-years-old and described by the patient as “quiet and submissive.” During the patient’s childhood, the mother was beaten and stabbed by her father, who is now and was described as “mean, violent, and very scary.” AC had not had any contact with her father, until three years prior to his death, when he apologized for his abuse. She has six siblings, with whom she has distant relationships.

#### Social history

The patient is a high school graduate with some college courses. She has been married twice and has three children, one of whom is “problematic.” She is supported by Social Security Disability Insurance benefits.

#### ADHD history

She admitted to being distractible during grade school and would frequently be dreamy and look out the window. She denied restlessness as well as motor or vocal tics.

#### Past medical history

The patient is status-post thyroidectomy, maintained on Synthroid. She suffers from migraine headaches, terrifying lucid dreams, and lactose intolerance.

#### Substance abuse history

She denied a history of alcohol dependence in the past. She denied drug abuse in the past, although she admitted to the use of marijuana and overusing prescription medications. The patient is a non-smoker.

#### Mental status examination

When initially seen, she was casually dressed and made good eye contact. She was oriented in all three spheres and was cooperative and pleasant. Her mood was described as extremely depressed. She had suicidal ideation, without plan. She had homicidal ideation, without plan. She denied auditory hallucinations, symptoms of OCD, delusions, or mania. She admitted to generalized anxiety as well as hyper-vigilance and nightly lucid nightmares, during which she felt “helpless.” She reported daily flashbacks of earlier traumas and admitted to a history of burning herself with hot water or a hot knife. She ate three meals a day and slept only 0 to 2 hours. Her thought processes were organized and linear.

#### Dream life

The patient was referred for individual therapy and group Dialectical Behavioral Therapy (DBT). She was started on prazosin at bedtime. Her initial Zoloft and Seroquel doses were increased. She continued to complain of symptoms of PTSD. Her mood was extremely depressed, but her sleep and appetite were fair. She was being seen in group and individual DBT. She had a dream about getting married again, which was interpreted by the psychiatrist as a possible sign of hopefulness, in contrast to her life-long dreams involving helplessness and hopelessness. Her mood was normal, and there was neither suicidal or homicidal ideation nor manic changes. Her appetite was normal, but she continued to complain of “nightmares.” She had been started on prazosin, with the dose increased from 2 mg to 6 mg at bedtime. She complained of problems with her children, but reported that her mood was essentially normal, and there was no evidence of suicidality, homicidality, or terrifying lucid dreams. She did, in fact, report a decreased intensity in her dreams, which was coincident with the recently prescribed and increased Adderall 20 mg in the morning. The patient complained of nightmares again, with a fair mood and no evidence of suicidal, homicidal, or manic symptoms. She continued individual and group DBT. She thought that prazosin was making her nightmares worse. In particular, she described lucid dreams of an unpleasant nature. These dreams, upon further interrogation, were “as if I were fully awake” and had a very primal content, involving rape and murder. Her prazosin, which she maintained was exacerbating her vivid, lucid, primal dreams, was decreased to 4 mg at bedtime. Prior to the next session, her visiting nurse stated that there was a need to decrease her prazosin further because of an increase in her heart rate (reflex tachycardia). Xanax was given at bedtime in an attempt to alleviate the lucid dreams with traumatic content. The patient then reported a lucid dream, wherein she was trying to dig out from behind a thick wall with a pick ax. She had opened up a hole in the wall, but could not get her entire body through this hole. Her psychiatrist interpreted the hole as a sign of hope and speculated that the self-built wall represented the patient’s mistrust and strong defense against expressing any feelings or impulses (a character trait adopted since early childhood). Additionally, the interpretation was advanced that the responsibility for the wall and its erection was a decision of the patient. The patient reported a depressed mood but no suicidal or homicidal ideation nor mania. She stated that she was having lucid sexual dreams again, in which she was “helpless.”

#### Treatment course

The patient reported a decrease in her head tremor and her mood was relatively normal. She reported that she was eating only one meal a day and had started taking KB220Z and noted decreased hunger on this nutraceutical. Off the Adderall, her concentration had decreased somewhat. She noted that while taking the KB220Z, her blood sugar had increased to 200, and she was placed on metformin, but that her bad lucid dreams had become somewhat happier. Her nutraceutical (KB220Z, which contained chromium) theoretically should not have increased her blood sugar. Nevertheless, her KB220Z was decreased to one pill in the morning and two at bedtime. She stated that the combination of metformin and KB220Z was associated with a blood sugar decrease from 135 to 123 mg/ml. She stated at this point that she was only taking one KB220Z in the morning and one at bedtime and felt much calmer. She also reported that the content of her dreams had become “much happier.” In addition, she said that she was no longer having nightmares or lucid dreams at all and that this had been the case for two months, which was coincident with the initiation of KB220Z. Her lucid dreams appeared to have satisfied the criteria of such dreams, since they were as if she were fully awake, were in vivid color and could be, to a certain extent, controlled by her voluntarily. Interestingly, the patient had missed the next few appointments because of transportation issues. She stated that during this time she had missed taking the KB220Z for three days and noted worsening lucid dreams, involving someone being raped.

She had missed a few days of KB220Z and stated that her lucid and traumatically terrifying dreams had returned. When she resumed taking three KB220Z a day, her vivid dreams persisted, but the content was not unpleasant. The patient expressed frustration with her visiting nurse. Her KB220Z continued to be prescribed at two tablets twice a day from the previous visit, and the patient reported that on a lower dose she had dreams of being raped, but that this constituted the first truly bad dream of a lucid nature on this regimen. She reported that her mood was depressed, that she had lost some weight, and that her dreams were normal on the four KB220Z a day, and that she was unable to remember any content, either pleasant or unpleasant. At this point, the patient wanted, because of an obsession about her weight and her resistance to following standard medical advice, to try a weight loss product known as Herbal Life. Because of possible, but unknown interactions with the KB220Z, it was decided to discontinue the latter, for the time being. After a 6-month period without KB220Z, the terrifying lucid dreams returned again. However, when she began the KB220Z again within 2 weeks, she reported no more terrifying lucid dreams. This patient has not had a terrifying dream for 12 months.

### Demographics

In an attempt to determine the possibility that the protracted effect of KB220z (as observed in Table 1) induced enhanced connectivity volume in brain regions linked to reward, the following rat experiments while not chronic may provide some.

## KB220Z Enhances Connectivity Volume in Rats

In order to obtain some information regarding the prolonged effect of KB220Z attenuating terrifying lucid dreams, we employed a well-researched electronic segmented rat brain atlas and specifically evaluated connectivity volume following the oral administration of KB220Z. In a previous study by Blum et al. [20], it was shown in rats that KB220Z considerably triggers, beyond placebo, seed areas of interest, involving the left nucleus accumbens, cingulate gyrus, anterior thalamic nuclei, hippocampus, pre-limbicandinfra-limbic loci. This reaction stimulated by KB220Z expresses noteworthy operative connectivity, augmented brain volume conscription (potentially neuroplasticity), and improved dopaminergic functionality throughout the brain reward circuitry. While these findings are observed in naïve rodents [20], we believe that this robust, yet selective response implies clinical relevance for victims of RDS, which show reductions in functional connectivity.

**Figure 1:**
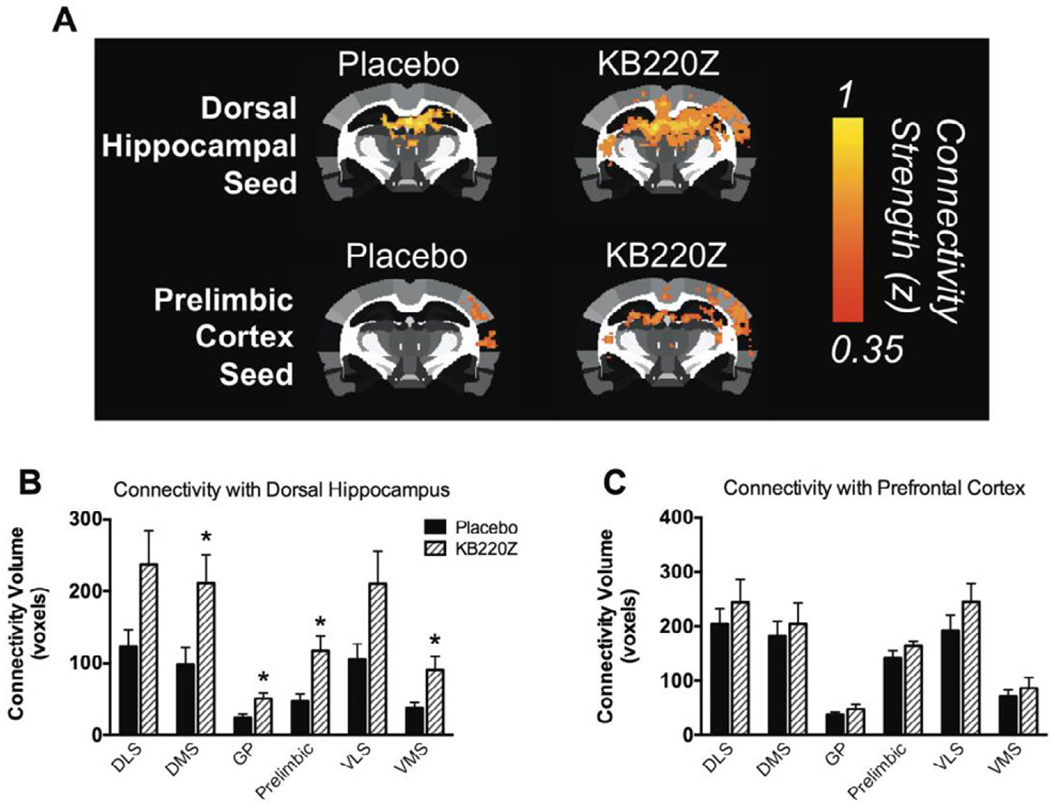
KB220Z increases the volume of regions showing connectivity with the hippocampus Connectivity volume was evaluated by employing a correlation threshold value of z = 0.3 to all participants and measuring the volume beyond this threshold. A) Functional connectivity maps showing connectivity with the hippocampus. KB220Z increased hippocampal connectivity. B) Connectivity volume of various brain regions with hippocampus (in voxels ± standard error; t-test *p < 0.05). C) Connectivity volume of the same regions in B with prefrontal cortex (prelimbic area). **Abbreviations:** DLS/DMS/VLS/VMS: dorsolateral, dorsomedial, ventrolateral, ventromedial striatum; GP: globus pallidus.

Utilizing the same methodology as Blum et al. [20] and others [21], we now report on the induction of enhanced connectivity volume when we seed the dorsal hippocampus [7]. We show versus placebo, significant effects in the following select brain regions of the basal ganglia: dorsmomedial, globus pallidus, pre-limbic, and ventromedial striatum. On the other hand, seeding the prelimbic cortex, the administration of KB220Z did not significantly increase connectivity in any of these brain regions, suggesting a selective regional effect. While we did not evaluate deeper structures like the pedunculopontine nucleus and visual cortical areas, also associated with lucid dreams (a subject of additional research), the selectively enhanced volume in the hippocampus may have clinical relevance [see Figure 1].

## Discussion

We do not yet understand the specific mechanism by which, in these four cases, three of whom were opiate/opioid addicts and diagnosed with ADHD, there was a protracted effect, involving the attenuation and eventually, perhaps, permanent elimination of what were often almost life-long terrifying, lucid dreams. However, the authors can only speculate that this surprising finding may have prolonged activity due to enhanced connectivity volume similar to that seen in our rat studies. The finding is, indeed provocative and, if replicable with a larger sample in an appropriate, double-blind, placebo-controlled study by independent investigators, could provide important insight into core RDS treatment and relapse prevention and long-term recovery.

The robust animal finding of enhanced connectivity volume, following the administration of KB220Z, compared to placebo, suggests, but does not prove increased neuroplasticity changes with potentially important clinical relevance for addicts [22, 23] showing reduced mesolimbic functional connectivity. In a related vein, Tomasi et al. [24] reported that, compared to neutral cues, food and cocaine cues increasingly activated the cerebellum as well as the orbitofrontal, inferior frontal, and premotor cortices and insula, while de-coupling the cuneus and default mode network (DMN). Theses authors found that these fMRI signals were proportional to striatal D2/D3 receptors. Surprisingly, cocaine and food cues also deactivated the ventral striatum and hypothalamus. Compared to food cues, cocaine cues produced lower activation in the insula and postcentral gyrus as well as less deactivation in the hypothalamus and DMN regions.

Stimulation in the cortical areas and cerebellum was augmented in comparison to the valence of the cues, and stimulation to food cues in somatosensory and orbitofrontal cortices also were augmented, in comparison to body mass. Lengthier contact to cocaine was related to decreased stimulation to both cues in the occipital cortex and cerebellum, which could suggest the reductions in D2/D3 receptors linked to chronicity of use. It is noteworthy that Meng et al. [25] showed that the injury to somato-sensory cortices before, rather than after morphine habituation, damaged the attainment of place preference suggesting the importance of this region in reward–like behavior.

The enhanced connectivity volume observed herein with KB220Z seems to support our earlier work in abstinent, human heroin addicts [7]. In that case, we found that compared to placebo, KB220Z induced an increase in the blood oxygenation level dependent (BOLD) response in caudate-accumbens-dopaminergic pathways, compared to the placebo, following 1-hour acute administration in abstinent, heroin addicts. The authors also found that KB220Z, compared to the placebo, increased functional connectivity in a putative system that incorporated the dorsal anterior cingulate, medial frontal gyrus, nucleus accumbens, posterior cingulate, occipital cortical regions, and cerebellum. The selectivity of brain regions (> 65 possibilities tested in this rat study) showing enhanced connectivity volume versus placebo, suggests that this finding is not rather widely distributed, but rather, seems to be linked to Reward Deficiency regions.

Interestingly, synaptic plasticity in the frontal lobe was found to be modulated by dopamine D1 receptors [15]. Gass et al. [26] found that the D2 selective blocker, haloperidol, significantly reduced functional connectivity between the substantia nigra and numerous further brain areas, specifically, the cingulate and prefrontal cortices, post-dorsal hippocampus, ventral pallidum, and motor cortex. Moreover, the haloperidol-induced focal changes in functional connectivity were found to be the most strongly associated with ascending dopamine projections. Furthermore, in support of dopamine D2 receptor involvement of enhanced connectivity volume found in the present study with KB220Z, Madularu et al. [27] reported that chronic administration of haloperidol reduced connectivity volume in awake, female rats.

While we do not yet know the particular origin for our prolonged findings with KB220Z, concerning the elimination of essentially life-long, terrifying, lucid dreams, in these four patients, three of whom were using buprenorphine/naloxone, it is not far-fetched to speculate that these results, in part, may be due to selectively enhanced connectivity volume in brain regions that have been previously linked to lucid dreams. Specifically, our findings in the rat study showed that by seeding the dorsal hippocampus, enhanced connectivity volume was seen in select brain regions previously linked to unwanted dreams such as the dorsomedial [28], globus pallidus [29], pre-limbic [30] and ventromedial striatum [31].

Importantly, Jones and Bonci [32] have suggested that changes in the strength of synaptic connections may have resulted in between the dopaminergic cells of the ventral tegmental area (VTA), in response to the use of several addictive drugs. Based on some studies to date [33], it has been hypothesized that drug-induced, synaptic plasticity may play a role in reward-related learning and addiction by modifying the fine tuning of dopaminergic cell firing. Further investigation in this critical area is necessary, and, if these initial studies are replicated, this result may have clinical relevance to patients suffering from lucid, terrifying dreams.

Following many years of intensive research showing many clinical benefits of a number of ingredient variants, over 30 studies [34] have been now published. To reiterate, it is important to realize that KB220Z not only provides precursor amino acids for important neurotransmitters (see above), it also promotes enkephalinase inhibition (d-phenylalanine), increases insulin receptor sensitivity (Chromium), benzodiazepine activation (passion flower) as well as COMT/ MAO-A activity inhibition (*Rhodiola rosea*). These effects are important in terms of balancing dopaminergic activity. To highlight a number of benefits published in peer-reviewed journals, the following represents clinical outcomes of KB220Z variants:

- Enhanced the brain enkephalin levels in rodents
- Reduced alcohol-seeking behavior in alcohol-loving C57/BL mice
- Pharmacogenetic conversion from alcohol-loving to alcohol-hating DBA mice
- In dependent humans, reported to reduce drug (opiates) and alcohol withdrawal symptomatology
- Reduced stress response, as measured by the skin conductance level (SCL) in recovering addicts
- Patients in treatment during recovery in a double-blind placebo-controlled study had significantly improved Physical and BESS Scores (behavioral, emotional, social, and spiritual)
- Following detox, compared to placebo patients (both alcoholics and cocaine abusers), there was a six-fold decrease in Against Medical Advice (AMA) rates
- Ten-month abstinent rate in driving under the influence (DUI) offenders 73% (alcoholics) and a 53% (cocaine dependent)
- Enhanced focus was demonstrated by healthy volunteers after 30 days of treatment
- Reduced cravings for alcohol, heroin, cocaine, and nicotine have been shown
- Reduced inappropriate sexual behavior demonstrated
- Reduced PTSD symptoms, such as protracted alleviation of lucid nightmares, have been reported
- Reduced widespread theta power in the anterior cingulate cortex in both alcoholics and heroin addicts
- Induction of enhanced alpha and low beta waves in anterior cingulate cortex of abstinent psychostimulant abusers using quantitative electroencephalography (qEEG)
- In abstinent heroin addicts, fMRI investigation, compared to placebo, a single dose increased resting state functional connectivity improvement of the prefrontal-cerebellar-occipital neural network and activation of the nucleus accumbens (NAc)
- In abstinent heroin addicts, compared to placebo, a single dose reduced hyperactivity of the putamen potentially decreasing unwanted emotionality
- In obese patients, carriers of the D2 receptors A1 allele (30–40% lowered D2 receptors) showed a significant Pearson correlation for enhanced compliance to days on treatment
- Double-blinded controlled studies and others have demonstrated positive effects on both craving attenuation and relapse prevention
- In rodents, fMRI analysis, compared to placebo, significantly activates the left nucleus accumbens, cingulate gyrus, anterior thalamic nuclei, hippocampus, prelimbic and infralimbic loci
- Significant functional connectivity, increased brain connectivity volume recruitment (potentially neuroplasticity), and dopaminergic functionality were found across the brain reward circuitry
- In an ADHD case study, using LORETA qEEG analysis, revealed after one dose, an increased frequency of each band in the anterior, dorsal and posterior cingulate regions, as well as the right dorsolateral prefrontal cortex during Working Memory (Steinberg et al. personal communication)

## Limitations

Based on the small number of patients investigated, we the authors, caution against drawing any definitive conclusions, until much larger, placebo-controlled studies are performed. With that stated, we are encouraged by these admittedly preliminary results indicating a protracted alleviation of terrifying, lucid dreams in RDS patients. Moreover, while we have some evidence in our rodent study related to enhanced connectivity volume in select brain regions following dorsal hippocampus seeding, we cannot firmly establish that similar changes have occurred in our small human sample.

## Conclusion

The authors have observed and found preliminary evidence in four cases, who exhibited prolonged, on-going effects of a putative pro-dopamine regulator, in the potential treatment of terrifying, lucid dreams. Such effects may be linked to the KB220Z’s ability to enhance the resting state functional connectivity in both rats [20] and abstinent heroin addicts [7], in addition to enhanced connectivity volume. One important possibility is that it may help balance dopamine dysregulation that has been observed in both ADHD [5] and in PTSD [35]. KB220Z has been shown to promote BOLD activity in the reward circuitry [7, 20]. Through optogenetic studies, it is clear that BOLD activation is tied to dopamine striatal neuronal firing [36]. Thus, the effects described herein for KB220Z could be working through dopamine activation. These findings require further investigative attention before definitive conclusions can be drawn.

## Figures and Tables

**Table 1:** Demographics of the four cases.

Subject	Gender	Age	Diagnosis without Lucid Dreams	Months Off	KB200Z Dream** Response
OB	M	47	ADHD, addiction	10 (mos.)	Complete Cessation
HK	M	38	ADHD, addiction	12 (mos.)	Complete Cessation
JC	F	32	ADHD, addiction	12 (mos.)	Complete Cessation
ACF	F	55	ADHD/PTSD, obesity	12 (mos.)	Complete Cessation

** In this sample, lucid dream content, prior to KB200Z, was uniformly unpleasant and/or terrifying in nature (e.g., being surrounded by snakes, drowning, being caught in a fire, etc.).
